# Interleukin-22 mitigates acute respiratory distress syndrome (ARDS)

**DOI:** 10.1371/journal.pone.0254985

**Published:** 2021-10-01

**Authors:** Sharven Taghavi, Olan Jackson-Weaver, Sarah Abdullah, Alanna Wanek, Robert Drury, Jacob Packer, Aaron Cotton-Betteridge, Juan Duchesne, Derek Pociask, Jay Kolls

**Affiliations:** 1 Department of Surgery, Tulane University School of Medicine, New Orleans, LA, United States of America; 2 Tulane University School of Medicine, Center for Translational Research in Infection and Inflammation, New Orleans, LA, United States of America; University of Alabama at Birmingham, UNITED STATES

## Abstract

**Background:**

The goal of this study was to determine if IL-22:Fc would Acute Respiratory Distress Syndrome (ARDS).

**Summary background data:**

No therapies exist for ARDS and treatment is purely supportive. Interleukin-22 (IL-22) plays an integral component in recovery of the lung from infection. IL-22:Fc is a recombinant protein with a human FC immunoglobulin that increases the half-life of IL-22.

**Study design:**

ARDS was induced in C57BL/6 mice with intra-tracheal lipopolysaccharide (LPS) at a dose of 33.3 or 100 ug. In the low-dose LPS group (LDG), IL-22:FC was administered via tail vein injection at 30 minutes (n = 9) and compared to sham (n = 9). In the high-dose LPS group (HDG), IL-22:FC was administered (n = 11) then compared to sham (n = 8). Euthanasia occurred after bronchioalveolar lavage (BAL) on post-injury day 4.

**Results:**

In the LDG, IL-22:FC resulted in decreased protein leak (0.15 vs. 0.25 ug/uL, p = 0.02). BAL protein in animals receiving IL-22:Fc in the HDG was not different. For the HDG, animals receiving IL-22:Fc had lower BAL cell counts (539,636 vs 3,147,556 cells/uL, p = 0.02). For the HDG, IL-6 (110.6 vs. 527.1 pg/mL, p = 0.04), TNF-α (5.87 vs. 25.41 pg/mL, p = 0.04), and G-CSF (95.14 vs. 659.6, p = 0.01) levels were lower in the BAL fluid of IL-22:Fc treated animals compared to sham.

**Conclusions:**

IL-22:Fc decreases lung inflammation and lung capillary leak in ARDS. IL-22:Fc may be a novel therapy for ARDS.

## Introduction

Acute Respiratory Distress Syndrome (ARDS) is a major clinical challenge worldwide. In the United States, ARDS is common, occurring in approximately 200,000 Americans per year [1, 2], with numbers likely to be increasing during the coronavirus disease of 2019 (covid-19) pandemic. ARDS has high morbidity and mortality, with death rates ranging up to 80% [3, 4]. For those that survive ARDS, there are significant long-term negative effects on quality of life, mental health, and neurocognitive function [5–7]. Current treatment remains primarily supportive, with ARDSnet lung-protective strategies and proning maneuvers improving survival, but no distinct therapeutic exists to reduce mortality [8, 9]. Extracorporeal Membrane Oxygenation (ECMO) and early neuromuscular blockade have also been proposed as treatments for ARDS, yet with conflicting results [9–11]. A novel therapy to treat ARDS has the potential to drastically decrease morbidity and mortality and this has never been more apparent than in the current coronavirus (covid-19) pandemic.

Interleukin-22 (IL-22) mediates epithelial repair in the injured lung. Most studies examining IL-22 have focused on its role of the injured lung after infection. Pre-clinical studies have shown that IL-22 has anti-fungal activity [12] and helps improve bacterial clearance [13–16]. IL-22 is required for normal repair of the injured lung after influenza infection [17] and mice treated with IL-22 show decreased inflammation and lung leak. These pre-clinical data suggest that IL-22 may have therapeutic potential in other forms of acute lung injury (ALI) [17]. IL-22 can signal through IL-10R2 receptors and IL-22Ra1 receptors. While the IL-10R2 receptor is widely expressed, IL-22Ra1 is limited predominantly to the epithelial cells of the skin, intestines, and lung. Whether the IL-22Ra1 receptor is expressed normally in the lung parenchyma is controversial [18–20]. Prior studies have shown that it is expressed in the alveoli after injury (20). Collectively, these studies have shown that IL-22 receptors are present on epithelial cells in the lung, and that stimulation of these receptors increases epithelial integrity as well as augmenting bronchial epithelial cell proliferation and inhibiting apoptosis [12, 14–17].

Studies on the role of IL-22 in the injured lung have been limited to infection models and no such published studies have examined its role in ALI or ARDS. Since IL-22 has been shown to have therapeutic potential by decreasing lung inflammation and injury in a murine model of H1N1 influenza [17], we hypothesized that the IL-22 protein given after acute lung injury (ALI) in mice would mitigate inflammation and repair ARDS-associated lung injury. To test this hypothesis, we used IL-22:Fc that prolongs IL-22 half-life in vivo [21].

## Methods

The study was carried out in strict accordance with the recommendations in the Guide for the Care and Use of Laboratory Animals of the National Institutes of Health. The protocol was approved by the Tulane University Institutional Animal Care and Use Committee (Protocol ID: 607). All surgery was performed under isoflurane anesthesia and all efforts were made to minimize suffering. All animals were monitored every 24 hours and each animal was examined for signs of distress or suffering such as hunched posture or respiratory distress. Any animals demonstrating suffering or distress were euthanized. The method of euthanasia was cervical dislocation and exsanguination under anesthesia.

### Acute lung injury and IL-22:Fc treatment

After approval from the Tulane University, Institutional Animal Care and Use Committee (protocol ID 607), equal numbers of male and female, 6–8 week old C57BL/6 mice (Charles River Laboratories, Cambridge, MA) were given ALI via intra-tracheally administered lipopolysaccharide (LPS). After obtaining appropriate depth of anesthesia using isoflurane, the high-dose LPS group (HDG) received 100 ug of LPS administered intra-tracheally. Approximately 30 minutes after LPS administration, 4 ug of IL-22:FC was administered via tail vein injection (n = 11), then compared to animals receiving sham injection (n = 8) with phosphate-buffered saline (PBS). Tail vein injection was used to deliver IL-22:Fc as prior studies have shown that this route of injection allows delivery of the injectate directly to the lungs in mice [22, 23]. In the low-dose LPS group (LDG), 33.3 ug of LPS was administered intra-tracheally. IL-22:FC was again administered at 30 minutes (n = 9) and compared to sham injected animals (n = 9). The Interleukin-22:Fc (IL-22:Fc) protein is a recombinant fusion protein (F-652) (Evive Biotech, Shanghai, China) with two human IL-22 molecules linked to the Fc portion of human immunoglobulin G2, which extends the half-life of the molecule. The half-life of IL-22 is 2 hours, while the half-life of the fusion protein in vivo is 3.02 days [21]. Human IL-22 binds and activates the mouse receptor. Dosage of IL-22:Fc was determined from prior in vitro studies [24].

### Evaluation of lung injury

Euthanasia and bronchoalveolar lavage (BAL) were carried out on post-injury day 4. After obtaining appropriate levels of anesthesia with inhaled isoflurane, the trachea was cannulated using a 26 gauge needle and BAL was performed with three successive washes using 1 mL of phosphate buffered saline (PBS). Next, a small segment of the left lower lobe was removed and saved for RNA isolation. Finally, 1 cc of 4% paraformaldehyde was injected to the lung for fixation.

The BAL fluid was then centrifuged at 500 x gravity for 5 minutes. Cells were obtained from the BAL after centrifuge and cell counts performed. Cells were then affixed to glass slides and stained with Wright’s stain. To quantify protein in the BAL supernatant, a Bradford protein assay (Bio-Rad Laboratories) was performed. Protein was quantified by measuring absorbance at 595 nm on a BMG Labtech FLUOstar Optima plate reader. In addition, the BAL supernatant was used to measure pro-inflammatory cytokines using a Milliplex Mouse Cytokine/Chemokine Magnetic Bead Panel (Millipore Sigma). The 32 cytokines measured included Eotaxin, Granulocyte Colony-Stimulating Factor (G-CSF), Granulocyte-Monocyte Colony-Stimulating Factor (GM-CSF), Interferon-γ (IFN-γ), Interleukin-1α (IL-1α), IL-1β, IL-2, IL-3, IL-4, IL-5, IL-6, IL-7, IL-9, IL-10, IL-12 (p40 segment), IL-12 (p70 segment), IL-13, IL-15, IL-17, Interferon-γ induced protein 10 (IP-10), keratinocyte chemoattractant (KC), leukemia inhibitory factor (LIF), lipopolysaccharide-induced CXC chemokine (LIX), monocyte chemoattractant protein-1 (MCP-1), macrophage colony-stimulating factor (M-CSF), monokine induced by gamma-interferon (MIG), MIP-1α, macrophage inflammatory protein-1β/CCL4 (MIP-1β), MIP-2, regulated upon activation, normal T-cell expressed and presumably secreted (RANTES), tumor necrosis factor-α (TNF-α), vascular endothelial growth factor (VEGF).Human IL-22 was measured using an IL-22 Human ELISA kit (ThermoFisher Scientific). Mouse IL-22 was measured using an IL-22 Mouse/Rat Quantikine ELISA kit (R&D Systems).

### Histopathological evaluation

Immediately after sacrifice on post-injury day 4, lung tissue from the right lower lobe was fixed in 4% paraformaldehyde and cut into sections. The sections were stained with hematoxylin and eosin. Lung injury induced by LPS was assessed by a blinded reviewer with a numerical scoring scale ranging from 0–4. Regions of lung injury in sections were scored for the extent of intimal thickening, alveolitis, and the presence of proteinaceous material in the alveolar space. Scoring system was as follows: 0) no pathology, 1) mild inflammation around airways, less than 25% of lung involved, 2) patch focal alveolitis, 26–50% of lung involved, 3) widespread inflammation encompassing 51–75% of the lung involved, 4) 76–100% lung involvement with inflammation and blood in alveolar spaces. Representative images were taken.

### Endothelial glycocalyx measurements

After sacrifice on post-injury day 4, paraformaldehyde-fixed lung segments were flash-frozen in Optimal Cutting Temperature (O.C.T) compound (Sakura) and sectioned on a cryostat. Sections were then blocked phosphate-buffered saline (PBS) supplemented with 1% BSA. Tissue was then stained with 23 μg/mL WGA and 23 μg/mL 4′,6-diamidino-2-phenylindole in PBS with 1% BSA for one hour at room temperature in the dark. Sections were then washed three times with PBS and covered with Fluoro-Gel mounting medium (Electron Microscopy Sciences). Glycocalyx and nuclei (4′,6-diamidino-2-phenylindole) were imaged on an Olympus BX51 fluorescence microscope under identical conditions. ImageJ software was used to quantify glycocalyx fluorescence intensity in the alveolar capillaries from a minimum of 20 regions of interest from 3 mice per condition.

### Immunofluorescence stains

After sacrifice on post-injury day 4, lung tissue was fixed in 4% paraformaldehyde in phosphate buffered saline (PBS) overnight. Paraformaldehyde-fixed lung segments were flash-frozen in Optimal Cutting Temperature (O.C.T.) compound (Sakura) and sectioned on a cryostat. Tissue was then blocked in 1% bovine serum albumin (BSA) in PBS for one hour. Tissue was then incubated overnight in primary antibody for IL-22Ra1 (Invitrogen, Carlsbad, CA, Ma5-24017) and E-cadherin (Novus Biologicals, Englewood, CO, NBP2-16258) diluted 1:100 in 1% BSA in PBS. When staining for phosphorylated signal transducer and activator of transcription 3 (STAT-3), the primary antibody used was Phospho-Stat3 XP rabbit monoclonal antibody (Cell Signaling Technology, Danvers, MA, 9145). Cells were then washed with PBS 3x. Cells were incubated with secondary antibody, goat anti-mouse Alexa Fluor 488 (1:500; Invitrogen, A28175) and goat anti-rabbit Alexa Fluor 555 (1:500, Invitrogen, A27039) diluted in 1% BSA in PBS along with 0.1 ug/ml of 4,6 diamidino-2phylindole (DAPI) (Sigma) for one hour, followed by three washes in PBS. Cells were then cover slipped with Fluoro Gel mounting medium and imaged on an Olympus BX51 fluorescence microscope. Fluorescence intensity was quantified using ImageJ.

### RNAseq

Lung tissue was homogenized in Trizol buffer (Life Technologies) and total RNA extraction was performed according to Trizol manufacturer’s instructions. Total RNA was used to perform RNA sequencing. RNA quantity and quality were assessed using NanoDrop and Agilent RNA ScreenTape with Agilent 4150 TapeStation system. SMART-Seq Stranded total RNA sample prep kit (Takara Bio USA, Inc.) was used for library preparation as specified in the user manual, followed by Agilent DNA 1000 kit validation with Agilent 4150 TapeStation system and quantification by Qubit 2.0 fluorometer. The cDNA libraries were pooled at a final concentration 1.2pM. Cluster generation and 1 x 75 bp single read single-indexed sequencing was performed by High-Output kit v2.5 (75 cycles) on Illumina NextSeq 550. Raw reads were processed and mapped. The gene transcription data was analyzed using Advaita Bioinformatics iPathway Guide (Ann Arbor, MI, https://www.advaitabio.com/ipathwayguide) was used to analyze gene expression. This software analysis tool implements the ‘Impact Analysis’ approach that takes into consideration the direction and type of all signals on a pathway, the position, role and type of every as described in previous literature [25–28]. The count matrix was rounded to the nearest integer and assessed for differential gene expression using DESeq2 (v.1.10.1) [29]. Gene expression was considered different when fold change (FC) was > 1.5 (upregulation) or < 0.667 (downregulation) and the adjusted p value was ≤ 0.05. Pathway analysis was conducted using the log2 (fold change) and adjusted p-values from DESeq2 with Ingenuity Pathways Analysis® (IPA®, QIAGEN Redwood City, CA, www.qiagen.com/ingenuity) using an adjusted p-value cut-off of 0.10 [30]. Pathway heat maps of normalized counts from DESeq2 were generated with the heatmap.2 function of the g-plots package [31]. For IL-22:Fc responsive genes, fold changes compared to sham groups are reported for each surgery group. GEO accession number is pending.

### Real-time quantitative reverse transcription PCR

RNA was isolated with Trizol (Invitrogen) and used as a template for reverse transcriptase (iScript RT supermix, Bio-Rad). mRNAs were quantified by real-time PCR with IQ Sybr Green Supermix (Bio-Rad), and normalized against *PPIA* mRNA as the internal control gene. Relative changes in expression were calculated using the ΔΔCt method as established in prior studies [32]. Primer sequences are listed in Table 1.

**Table 1:** List of primer sequences used for quantitative PCR.

mRNA	Forward Primer	Reverse Primer
*PPIA*	5’-CCCACCGTGTTCTTCGACATT-3’	5’-GGACCCGTATGCTTTAGGATGA-3’
Tenascin C	5’- ACGGCTACCACAGAAGCTG-3’	5’-ATGGCTGTTGTTGCTATGGCA -
Collagen, type 1, alpha 1	5’-GCTCCTCTTAGGGGCCACT -3’	5’-CCACGTCTCACCATTGGGG -3’
Collagen, type 1, alpha 2	5’-GTAACTTCGTGCCTAGCAACA -3’	5’-CCTTTGTCAGAATACTGAGCAGC -3’
Collagen, type 6, alpha 3	5’-AAGGACCGTTTCCTGCTTGTT -3’	5’-GGTATGTGGGTTTCCGTTGAG -3’

### Statistical analysis

Normal distribution was tested by Shapiro- Wilk test. Student’s t-test and Mann-Whitney test were used where appropriate. Values are presented as means ± standard error and median with interquartile range where appropriate. A p-value of less than 0.05 was considered significant for all tests.

## Results

### Cell counts measured in BAL

**Fig 1:**
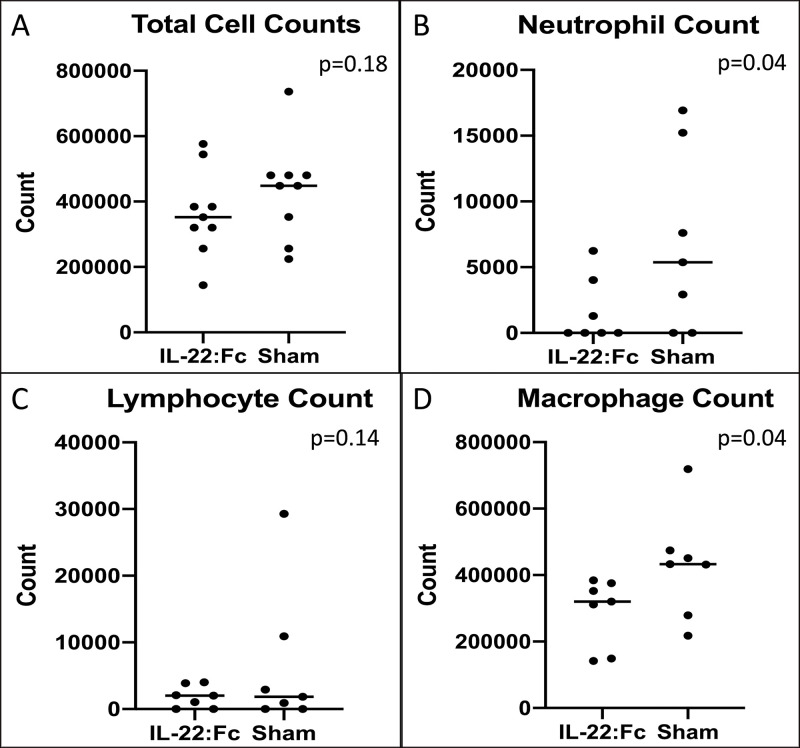
Cellular influx after low-dose LPS lung injury. Mice with low-dose LPS injury have decreased cellular influx of B) neutrophils and D) macrophages into the lungs when treated with IL-22:Fc as shown in BAL cell counts. There was no difference seen in A) total cell counts and C) lymphocyte counts.

To examine the degree of inflammatory cell influx in high dose injury animals, we compared cell counts between IL-22:Fc treated animals and sham animals. Cell counts for low-dose LPS injured animals are shown in Fig 1. As shown in Fig 1A total cell counts were not different in IL-22:Fc treated when compared to sham (364,444 vs. 433,889 cells, p = 0.16). Neutrophil count was lower in the IL-22:Fc treated (1,653 vs. 6,869 cells, p = 0.04) as shown in Fig 1B. Lymphocyte count was not different in IL-22:Fc treated (1,864 vs. 6,556 cells, p = 0.14), however, macrophage count was lower (290,611 vs. 429,262 cells, p = 0.04) as shown in Fig 1C and 1D.

**Fig 2:**
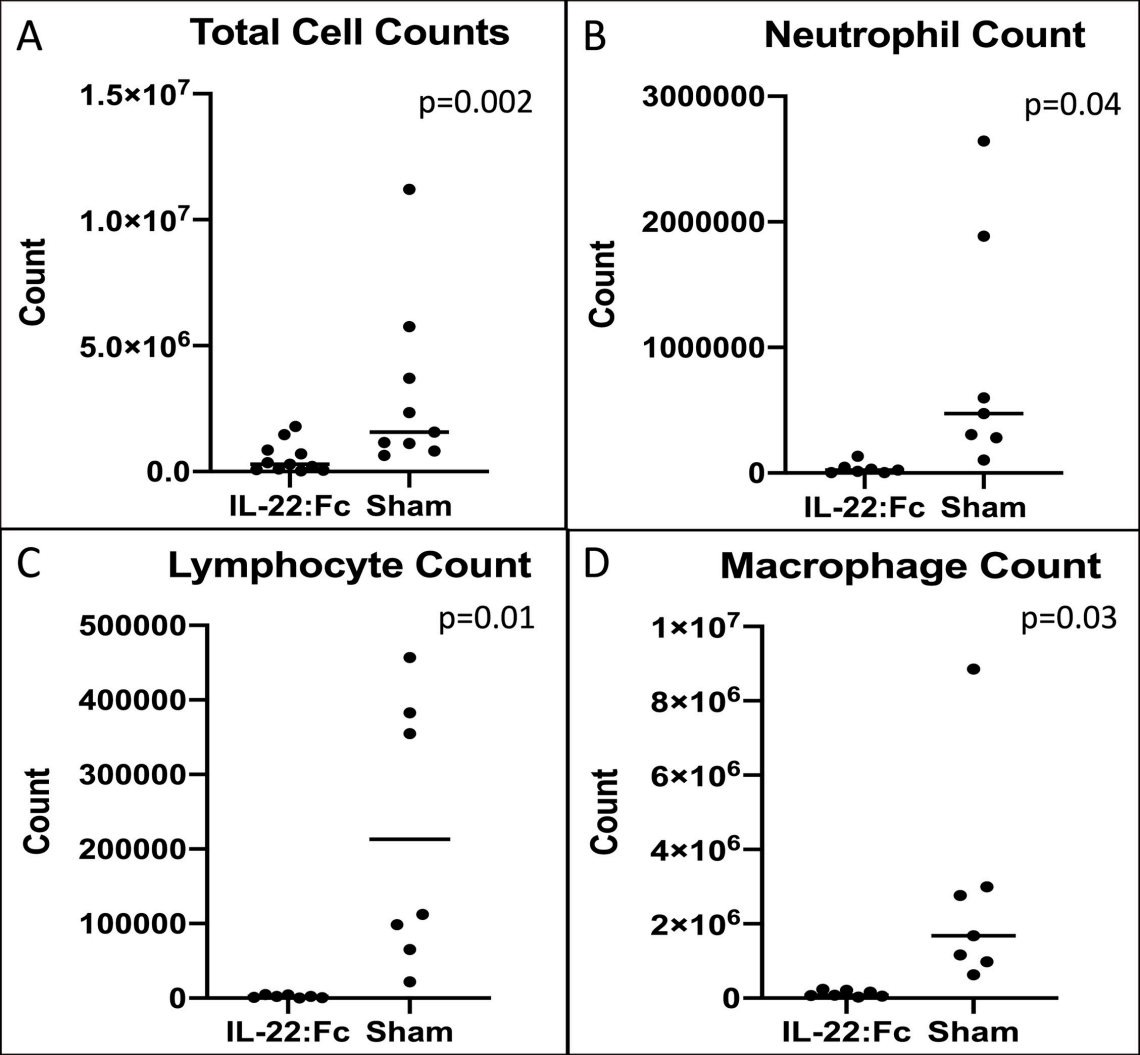
Cellular influx after high-dose LPS lung injury. Mice with high-dose LPS injury have decreased cellular influx into the lungs when treated with IL-22:Fc as shown in BAL cell counts. IL-22:Fc treated mice have decreased A) total cell counts, B) neutrophil counts, C) lymphocyte counts, and D) macrophage counts.

A comparison of cell counts for high-dose LPS injury is shown in Fig 2. Mice treated with IL-22:Fc had lower total cell counts (5.40 x 10^5^ vs. 3.15 x 10^6^ cells, p = 0.02), lower neutrophil counts (3.69 x 10^4^ vs. 8.99 x 10^5^ cells, p = 0.04), lower lymphocyte counts (2,163 vs. 213,225 cells, p = 0.01), and lower macrophage counts (1.21 x 10^5^ vs. 2.72 x 10^6^ cells, p = 0.03).

### BAL inflammatory mediators

To examine the degree of inflammation in the lungs after IL-22:Fc, we compared inflammatory mediators in BAL fluid of treated and untreated animals. A comparison of all inflammatory mediators measured in the BAL of mice with low-dose LPS injury is shown in Table 2. There was no difference in amount of any measured inflammatory mediators when comparing the IL-22:Fc treated to sham animals.

**Table 2:** A comparison of IL-22:Fc treated and sham animals after acute lung injury with low dose LPS.

Cytokine	IL-22:Fc Treated (pg/mL)	Sham (pg/mL)	p-value
Interleukin-1α	61.31 ± 12.36	47.00 ± 9.64	0.19
Interleukin-1β	0.01 ± 0.01	0.01 ± 0.01	0.50
Interleukin-2	6.77 ± 1.75	3.37 ± 1.11	0.06
Interleukin-3	0.00 ± 0.00	0.00 ± 0.00	0.99
Interleukin-4	0.00 ± 0.00	0.00 ± 0.00	0.99
Interleukin-5	0.00 ± 0.00	0.19 ± 0.19	0.17
Interleukin-6	0.95 ± 0.62	2.39 ± 1.47	0.19
Interleukin-7	0.00 ± 0.00	0.00 ± 0.00	0.99
Interleukin-9	154.50 ± 37.46	120.40 ± 37.64	0.27
Interleukin-10	19.91 ± 6.56	13.60 ± 6.53	0.25
Interleukin-12 (p40)	6.87 ± 1.88	5.83 ± 1.96	0.35
Interleukin-12 (p70)	0.00 ± 0.00	0.00 ± 0.00	0.99
Interleukin-13	2.69 ± 0.93	1.35 ± 0.88	0.16
Interleukin-15	0.00 ± 0.00	0.00 ± 0.00	0.99
Interleukin-17	0.40 ± 0.06	0.45 ± 0.08	0.32
Tumor Necrosis Factor-α	3.01 ± 1.79	3.01 ± 1.76	0.50
Eotaxin	2.59 ± 1.93	7.96 ± 3.65	0.11
Interferon-γ	1.15 ± 0.42	2.28 ± 1.17	0.19
Granulocyte Colony-Stimulating Factor	23.49 ± 9.15	33.76 ± 9.96	0.23
Granulocyte-Macrophage Colony-Stimulating Factor	0.00 ± 0.00	0.00 ± 0.00	0.99
Interferon-γ-Induced Protein-10	12.94 ± 3.54	25.88 ± 9.82	0.12
Keratinocyte Chemoattractant/Growth Regulated Oncogene	9.11 ± 2.76	10.72 ± 2.71	0.34
Monocyte Chemoattractant Protein	0.00 ± 0.00	0.00 ± 0.00	0.99
Macrophage Inflammatory Protein-1α	15.62 ± 2.03	19.89 ± 11.37	0.19
Macrophage Inflammatory Protein-1β	0.00 ± 0.00	2.15 ± 2.15	0.17
Macrophage Inflammatory Protein-2	20.45 ± 10.88	11.81 ± 5.57	0.25
Monocyte Induced by Interferon-γ	10.52 ± 4.73	26.22 ± 14.25	0.16
Macrophage Colony-Stimulating Factor	0.00 ± 0.00	0.00 ± 0.00	0.99
C-X-C Motif Chemokine 5 (LIX)	9.27 ± 6.04	0.76 ± 0.76	0.09
Vascular Endothelial Growth Factor	3.01 ± 0.50	3.17 ± 1.45	0.42

**Fig 3:**
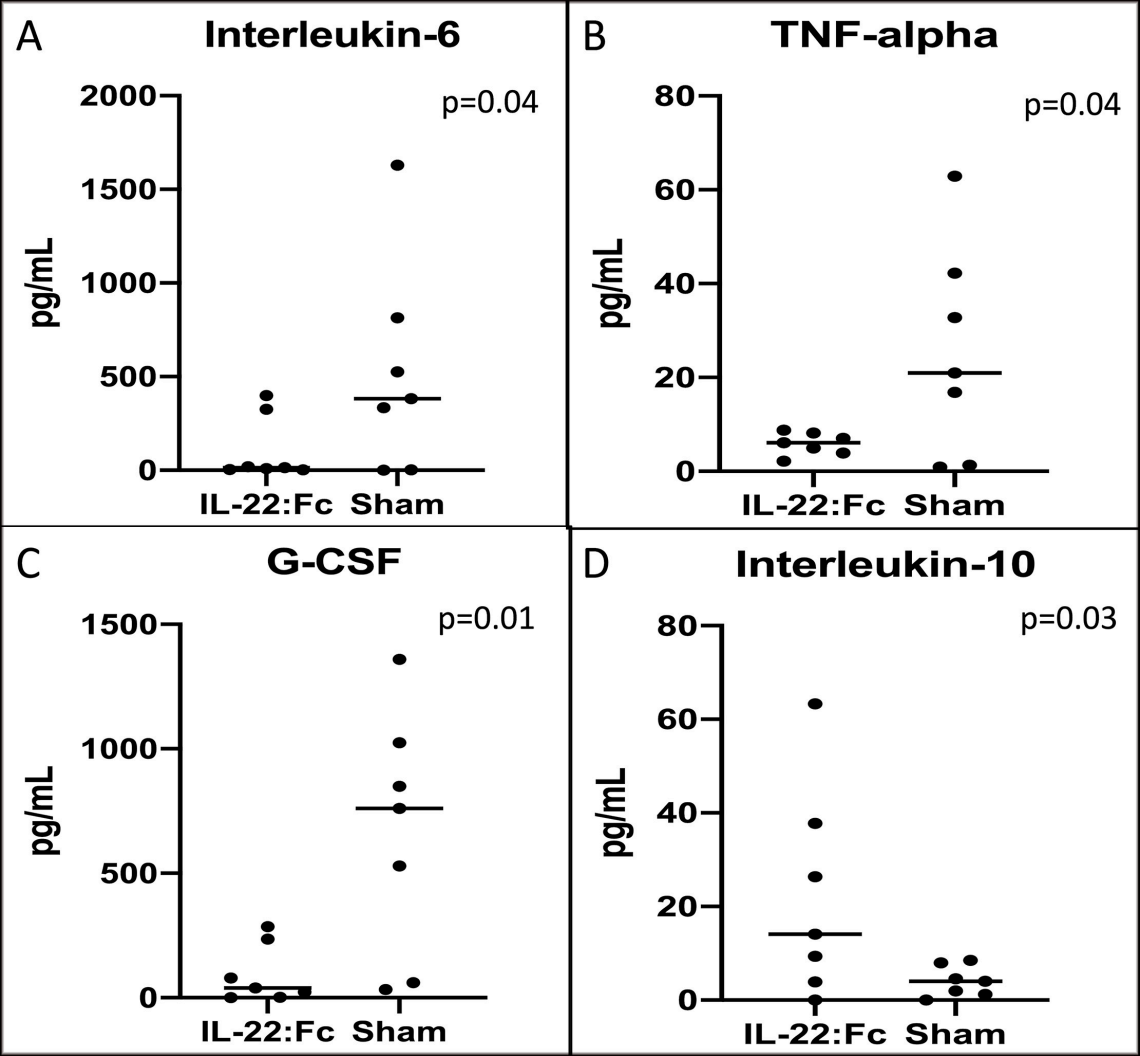
Measures of inflammatory mediators after high-dose LPS lung injury. Mice with high-dose LPS injury have decreased inflammation in the lungs when treated with IL-22:Fc as shown in BAL inflammatory mediators. IL-22:Fc treated mice have decreased A) Interleukin-6, B) TNF-alpha, C) G-CSF, and D) Interleukin-10.

Inflammatory mediators in high-dose LPS injured animals are shown in Fig 3. IL-6 (110.6 vs. 527.1 pg/mL, p = 0.04), TNF-α (5.87 vs. 25.41 pg/mL, p = 0.04), and G-CSF (95.14 vs. 659.6, p = 0.01) levels were lower in the BAL fluid of IL-22:Fc treated animals compared to sham controls. Interleukin-10 levels in BAL fluid were higher in IL-22:Fc treated (22.10 vs. 4.05 pg/mL, p = 0.03). A summary of all other cytokines measured in the multiplex assay is shown in Table 3. IL-1α, IL-2, IL-5, IL-9, IL-12, IL-15, and M-CSF were found to be lower in the IL-22:Fc treated.

**Table 3:** A comparison of IL-22:Fc treated and sham animals after acute lung injury with high dose LPS.

Cytokine	IL-22:Fc Treated (pg/mL)	Sham (pg/mL)	p-value
Interleukin-1α	69.39 ± 5.91	28.14 ± 5.40	<0.001
Interleukin-1β	3.48 ± 1.25	10.05 ± 3.26	0.04
Interleukin-2	7.33 ± 0.83	0.26 ± 0.45	<0.001
Interleukin-3	1.07 ± 0.02	1.05 ± 0.06	0.37
Interleukin-4	1.09 ± 0.09	0.90 ± 0.12	0.10
Interleukin-5	7.86 ± 1.14	0.29 ± 0.29	<0.001
Interleukin-7	1.75 ± 0.18	2.14 ± 1.16	0.37
Interleukin-9	311.8 ± 38.17	79.57 ± 15.47	<0.001
Interleukin-12 (p40)	7.13 ± 1.89	3.27 ± 1.04	0.049
Interleukin-12 (p70)	1.95 ± 1.00	2.88 ± 0.93	0.19
Interleukin-13	10.56 ± 1.49	7.92 ± 1.92	0.15
Interleukin-15	6.10 ± 1.02	1.85 ± 0.57	0.002
Interleukin-17	2.28 ± 0.56	12.34 ± 4.67	0.03
Eotaxin	4.63 ± 2.43	21.59 ±	0.09
Interferon-γ	8.08 ± 2.18	103.9 ± 92.04	0.16
Granulocyte-Macrophage Colony-Stimulating Factor	0.00 ± 0.00	0.38 ± 0.38	0.17
Interferon-γ-Induced Protein-10	118.40 ± 43.72	616.90 ± 171.1	0.01
Keratinocyte Chemoattractant/Growth Regulated Oncogene	19.54 ± 3.08	35.89 ± 12.54	0.11
Monocyte Chemoattractant Protein	14.66 ± 7.16	34.20 ± 15.08	0.13
Macrophage Inflammatory Protein-1α	45.99 ± 7.56	73.96 ± 15.40	0.07
Macrophage Inflammatory Protein-1β	19.42 ± 7.63	70.89 ± 21.44	0.02
Macrophage Inflammatory Protein-2	28.63 ± 5.43	16.54 ± 7.99	0.11
Monocyte Induced by Interferon-γ	100.60 ± 35.47	906.00 ± 295.40	0.01
Macrophage Colony-Stimulating Factor	2.89 ± 1.02	1.02 ± 0.41	0.049
C-X-C Motif Chemokine 5 (LIX)	0.00 ± 0.00	0.00 ± 0.00	0.99
Vascular Endothelial Growth Factor	12.18 ± 3.03	12.64 ± 5.22	0.47

### Protein leak and histopathology scores

To examine the degree of lung leak and lung damage, we measured BAL protein levels and compared histopathology scores. After low-dose LPS injury, BAL protein in animals receiving IL-22:Fc was lower than sham animals (0.15 vs. 0.25 ug/uL, p = 0.03). A comparison of histopathology scores among animals with low dose LPS injury did not show any difference in histopathology scores.

**Fig 4:**
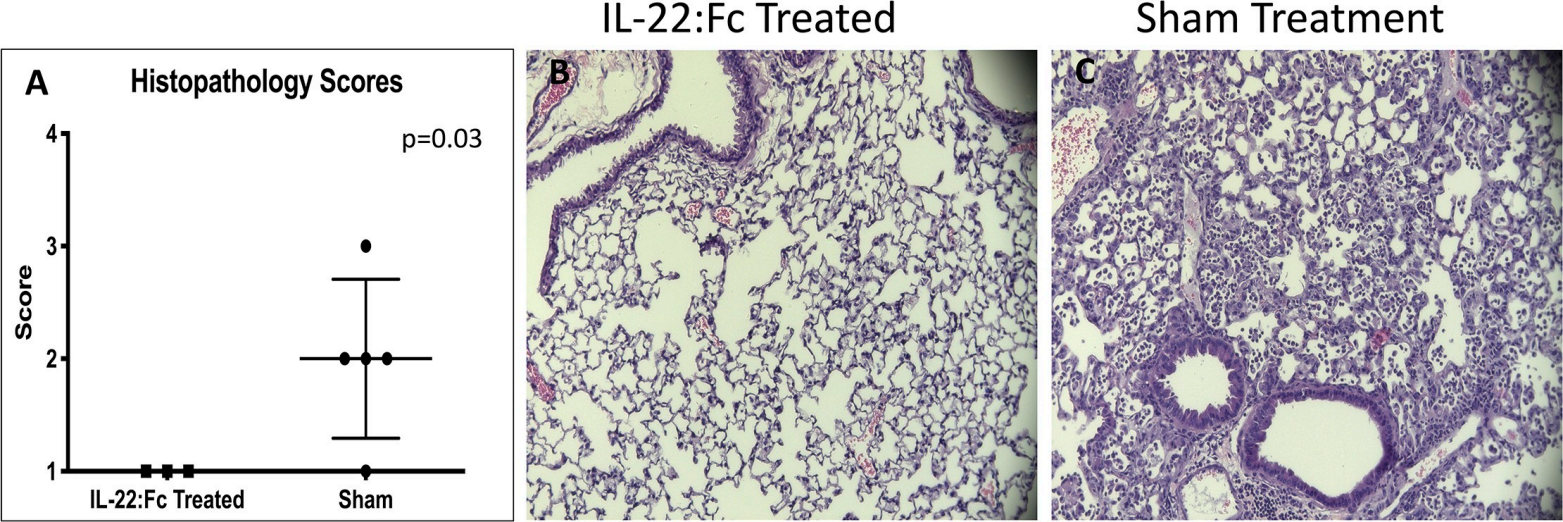
Comparison of histopathological scores in high-dose LPS lung injured mice. Mice with high-dose LPS injury have less severe damage to the lungs when treated with IL-22:Fc as seen with histopathology scores graded by a blinded reviewer (A). Representative images of lung tissue are shown in B) IL-22:Fc treated and C) Sham animals.

After high-dose LPS injury, BAL protein in animals receiving IL-22:Fc was not different compared to sham (0.55 vs. 0.38 ug/uL, p = 0.18). A comparison of histopathology scores of high-dose LPS inured animals (Fig 4A) showed that IL-22:Fc treated animals had less severe injury scores (1.0 vs. 2.0, p = 0.03). Representative images of IL-22:Fc treated and sham animals are shown in Fig 4A and 4B.

### Glycocalyx degradation

**Fig 5:**
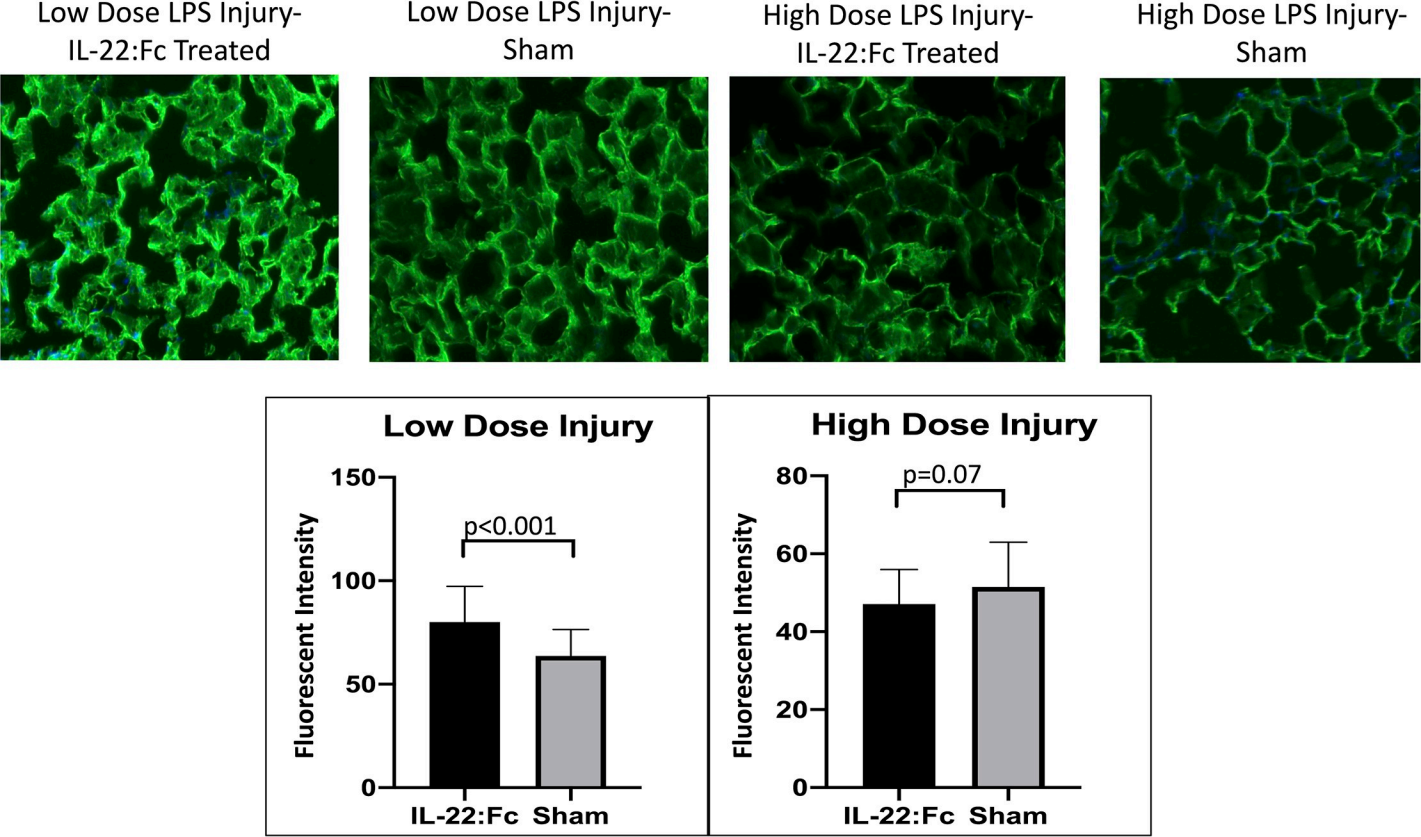
Comparison of endothelial glycocalyx staining intensity. IL-22:Fc treated mice have improved preservation of the endothelial glycocalyx in alveolar capillaries as compared to sham animals. Endothelial glycocalyx staining intensity was increased in the alveolar capillaries in IL-22:Fc treated mice after low-dose LPS injury. Endothelial glycocalyx staining intensity was not different for IL-22:Fc treated mice in high-dose LPS injury.

To determine if IL-22:Fc helps maintain the glycocalyx layer in the endothelium of alveolar capillaries, we measured endothelial glycocalyx intensity as seen in Fig 5. In the low dose LPS injury group, IL-22:Fc resulted in greater intensity of the glycocalyx (80.0 vs. 63.7 Arbitrary Units, p<0.001) after LPS injury. Representative images are shown in Fig 5.

In the high dose LPS injury group, there was no difference in glycocalyx intensity when comparing IL-22:Fc treated with sham.

### Exogenous vs. endogenous IL-22

**Fig 6:**
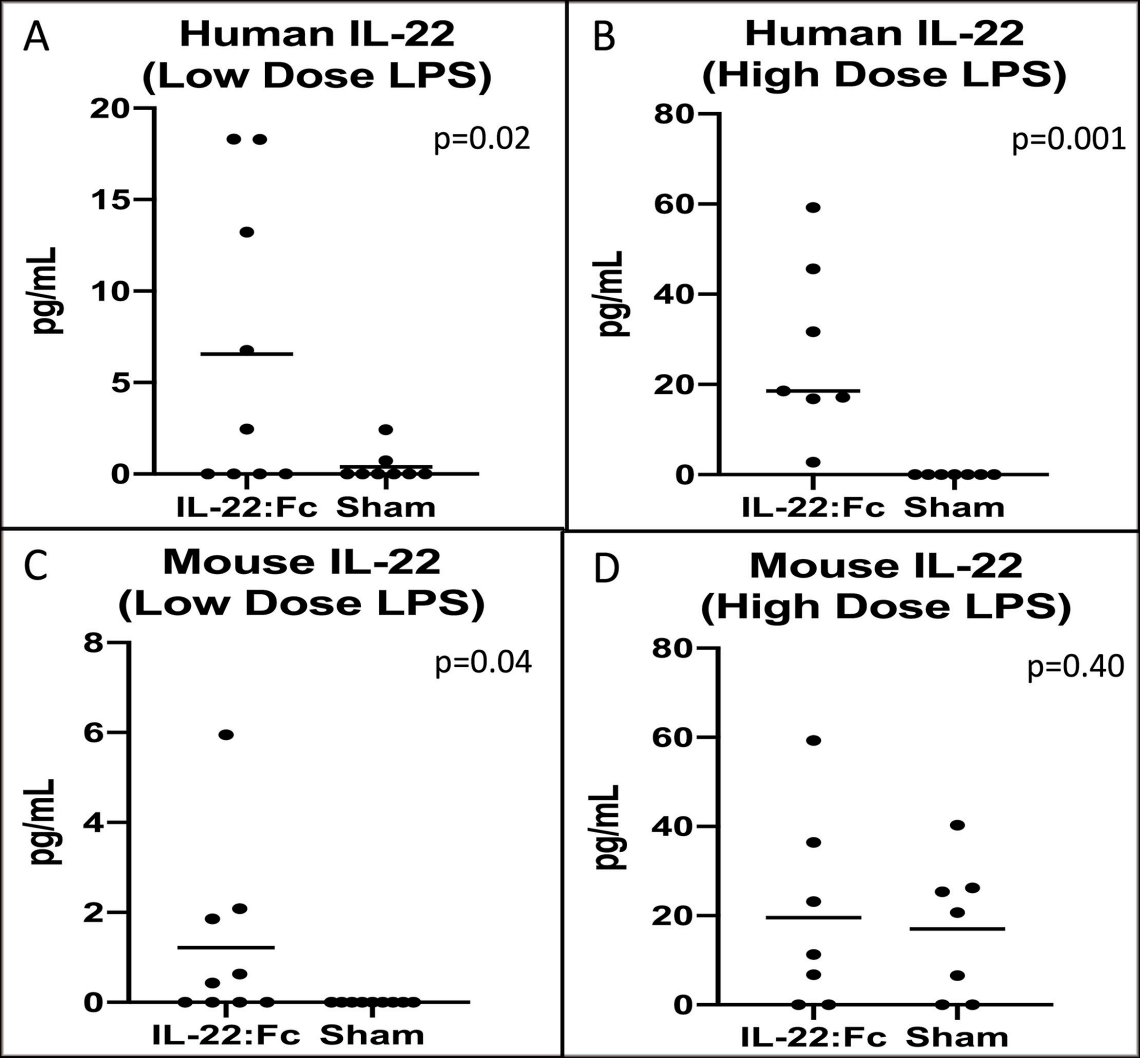
A comparison of exogenous and endogenous Interleukin-22 in the lungs. Because fk-652 is a human protein, we are able to differentiate between exogenous IL-22:Fc and endogenous IL-22:Fc. Treatment with IL-22:Fc appears to result in increased endogenous mouse IL-22 (C). Exogenous human IL-22 was detected in the BAL of treated mice (A and B) demonstrating that exogenous IL-22:Fc is reaching the lung. D) Endogenous mouse IL-22:Fc was not increased in IL-22:Fc treated after high-dose LPS injury.

To determine if the effect on the lungs was due to exogenous IL-22:Fc or endogenous IL-22, we measured human and mouse IL-22 in the BAL of both high and low dose LPS injured animals. As shown in Fig 6, there was higher levels of human IL-22 in the IL-22:Fc treated in both low dose-LPS (6.56 vs. 0.40 pg/mL, p = 0.02) (Fig 6A) and high-dose (27.41 vs. non-detectable pg/mL) (Fig 6B) injured animals. Endogenous mouse IL-22 levels in the low-dose LPS injury group was higher in the IL-22:Fc treated (1.22 vs. non-detectable pg/mL, p = 0.04) (Fig 6C). However, endogenous IL-22 was not different in the high-dose LPS injury animals treated with IL-22:Fc (19.57 vs. 17.02 pg/mL, p = 0.40) (Fig 6D).

### Examining the role of the IL-22Ra1 receptor and STAT3

**Fig 7:**
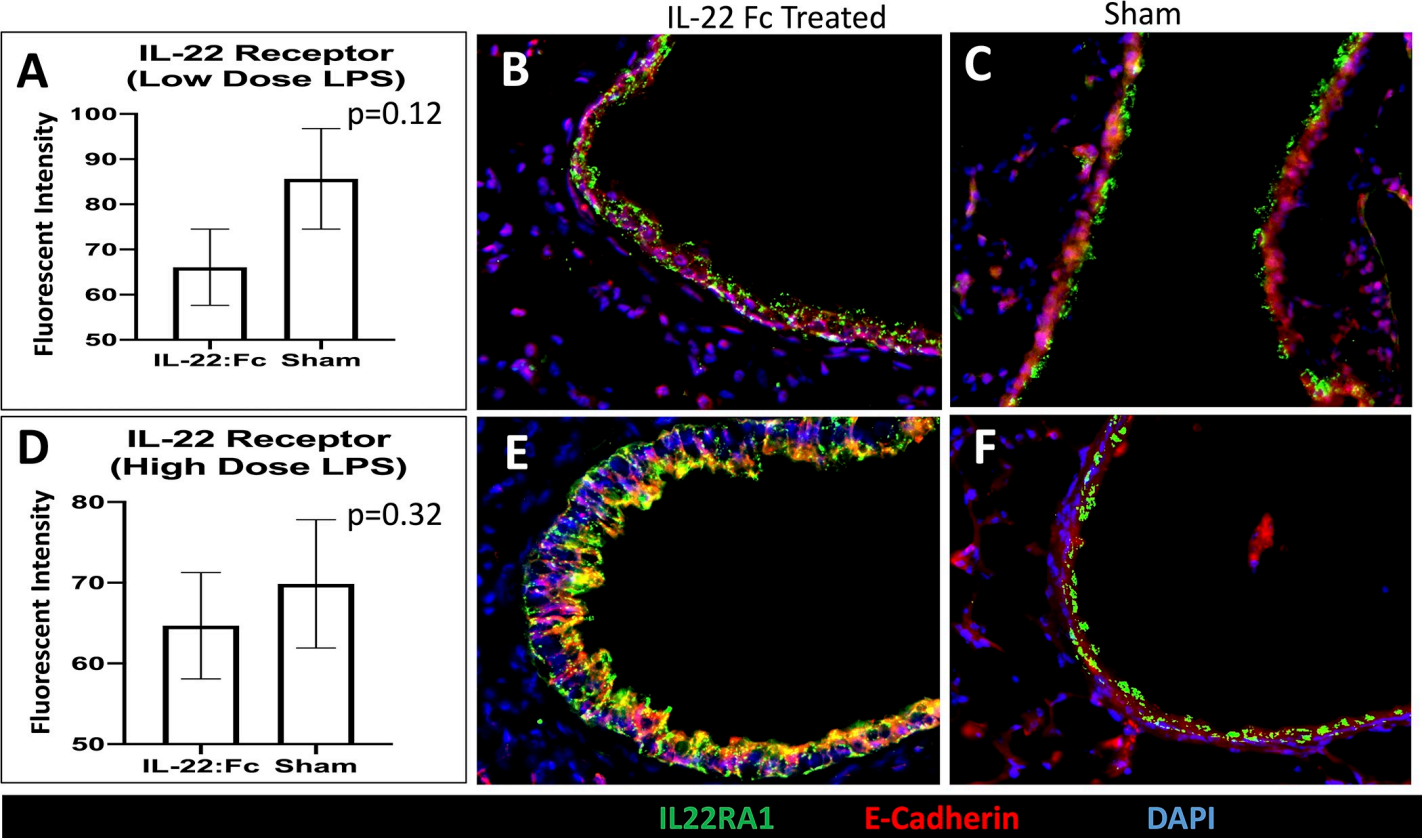
A comparison of the amount of IL-22 receptor in low- and high-dose LPS injured mice. The amount of IL-22Ra1 receptor was not different in the fk-652 treated animals in both low- and high-dose LPS injury (A and D). Representative images of the airways are shown in low-dose LPS injured for B) IL-22:Fc treated and C) sham animals. Representative images of the alveoli is shown in high-dose LPS injured for B) IL-22:Fc treated and C) sham animals.

To examine the role of the IL-22Ra1 receptor, we examined expression of the receptor on the airway. Expression of the IL-22Ra1 receptor was not significantly different in IL-22:Fc treated compared to sham (66.08 vs. 85.64 Fluorescence Intensity, p = 0.12) after low dose LPS injury (Fig 7A). Representative images after immunofluorescence staining for IL-22Ra1, E-cadherin, and DAPI is shown for IL-22Ra1 treated (Fig 7B) and sham (Fig 7C).

After high-dose injury, expression of the IL-22Ra1 receptor was not significantly different in IL-22:Fc treated compared to sham (64.68 vs. 69.87 Fluorescence Intensity, p = 0.32) (Fig 7D). Representative images after immunofluorescence staining for IL-22Ra1, E-cadherin, and DAPI is shown for IL-22Ra1 treated (Fig 7E) and sham (Fig 7F).

**Fig 8:**
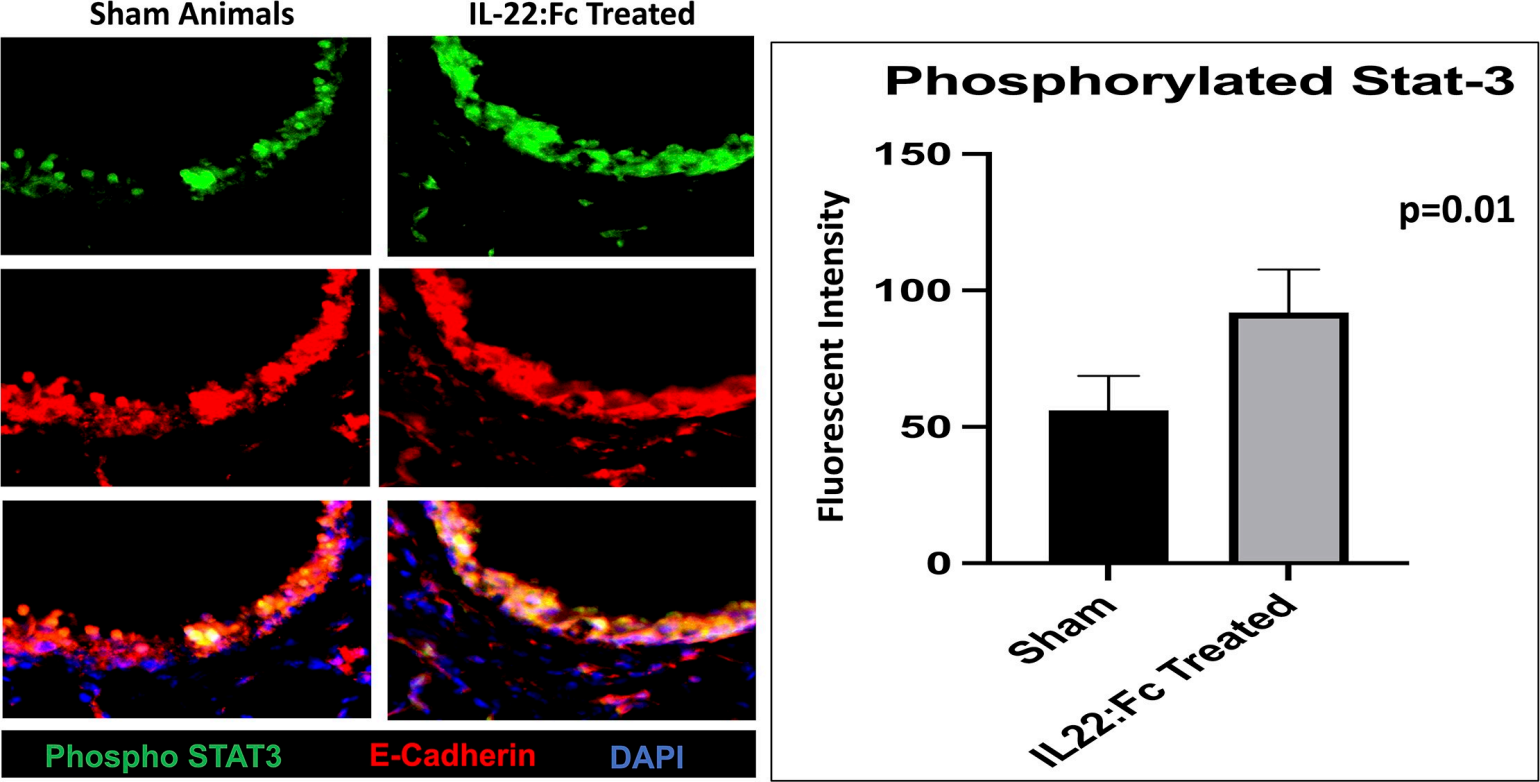
A comparison of the amount of phosphorylated-STAT-3 in low-dose LPS injured mice. The amount of phosphorylated-STAT-3 was not higher in the fk-652 treated animals in low-dose LPS injury (A and D). Representative images of the airways are shown in low-dose LPS injured for IL-22:Fc treated and sham animals. Representative images of the alveoli is shown in low-dose LPS injured for IL-22:Fc treated and sham animals.

To examine the role of pulmonary STAT-3 activation, we examined the amount of phosphorylated-STAT-3 in the airways in IL-22:Fc treated and sham animals after low-dose LPS injury. Animals treated with IL-22:Fc had higher amounts of phosphorylated-STAT-3 when compared to sham treated animals (91.9 vs. 56.1 AU, p = 0.01). Representative images after immunofluorescence staining for phosphorylated-STAT-3, E-cadherin, and DAPI is shown for IL-22Ra1 treated and sham (Fig 8).

### RNAseq analysis

**Fig 9:**
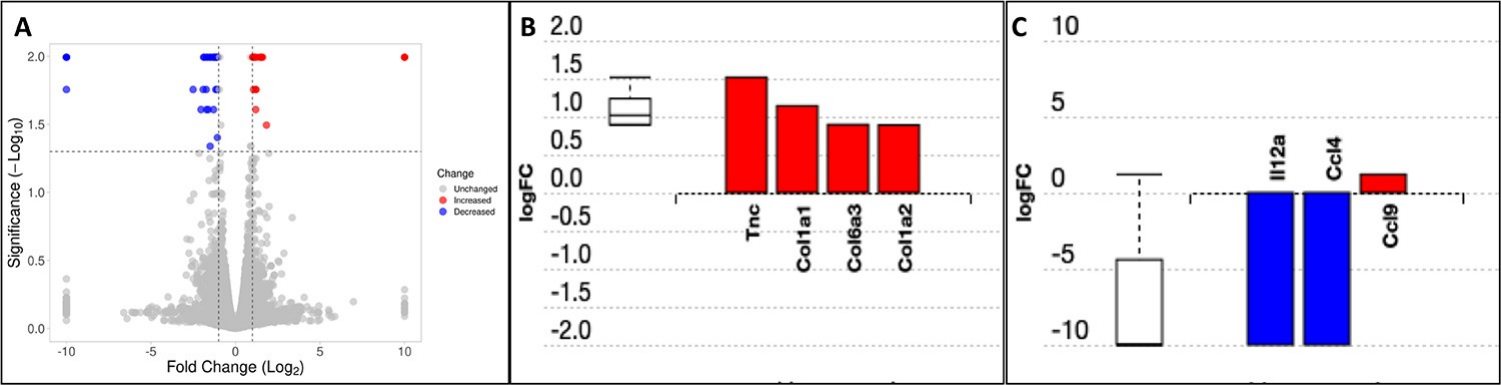
Gene expression differences in IL-22:Fc treated animals. Animals treated with IL-22:Fc after high-dose LPS injury have increased transcription of extracellular matrix components and decreased transcription of Interleukin-12a (IL12a) and Macrophage Inflammatory Protein-1β (CCL4). RNAseq data demonstrating A) volcano plot, B) increased expression of tenacin C, Tenascin C (Tnc), collagen, type I, alpha 1 (COL1a1), collagen, type VI, alpha 3 (Col6a3), and collagen, type I, alpha 2 (Col1a2), and C) decreased expression of IL-12a and CXCL4 genes for IL-22:Fc treated.

Pathway analysis of gene expression showed that the cytokine-cytokine receptor pathway was significantly different in IL-22:Fc treated animals after high-dose LPS injury (Fig 9). Differentially expressed pathway genes for extracellular matrix-receptor interactions were also different between groups (Fig 9B). Tenascin C (Tnc), collagen, type I, alpha 1 (Col1a1), collagen, type VI, alpha 3 (Col6a3), and collagen, type I, alpha 2 (Col1a2) expression was increased with IL-22:Fc treatment (p = 0.003). IL-22:Fc treatment resulted in a decrease in Interleukin-12a and Macrophage Inflammatory Protein-1β (CCL4) transcription (p = 0.01).

**Fig 10:**
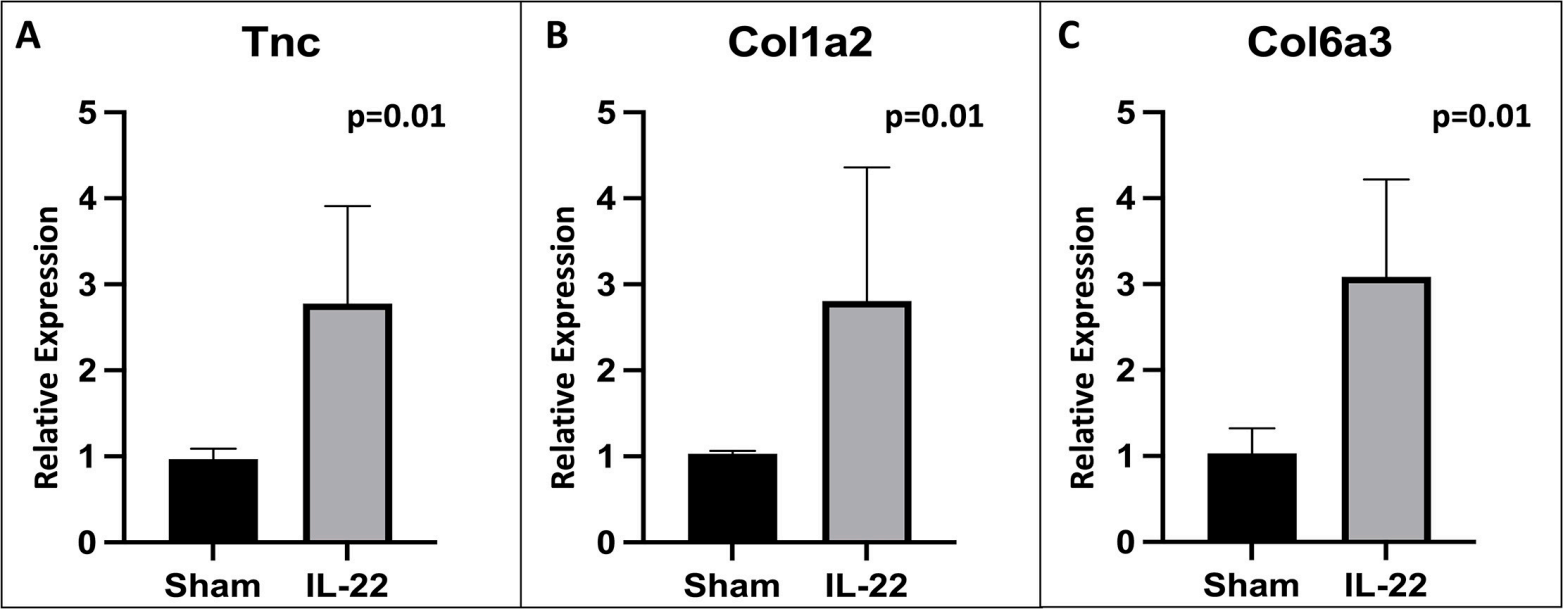
Qualitative PCR showing increased expression of extracellular matrix components in IL-22:Fc treated animals. Increased expression of A) Tenascin C (Tnc), B) collagen, type I, alpha 2 (Col1a2) and C) collagen, type VI, alpha 3 (Col6a3) in IL-22:Fc treated animals as compared to sham.

To confirm our RNAseq findings of extracellular matrix components, quantitative PCR was performed for transcripts of interest. This confirmed increased expression of Tnc (Fig 10A), Col1a2 (Fig 10B), and Col6a3 (Fig 10C) in IL-22:Fc treated animals. Col1a1 expression was not found to be different by PCR (1.02 vs. 3.82 relative expression, p = 0.13).

## Discussion

Interleukin-22 is integral to the repair of the injured lung and helps clear viral, bacterial, and fungal infection from the lung [12, 14, 15, 17, 20]. Whether f-652 can play a therapeutic role in ALI/ARDS has not been examined. While IL-22:Fc can have some pro-inflammatory properties, given prior studies have shown that it is beneficial in infectious lung disease, we hypothesized that the anti-inflammatory properties would help alleviate acute lung injury. Currently, ARDS is a disease state with significant morbidity and mortality, with no therapeutic having shown clinical benefit [33, 34]. In this study, we set out to determine if, IL-22:Fc, a human fusion protein with increased half-life can be used as a novel therapy in a sterile model of murine ARDS. A Phase 1 trial has already been conducted on IL-22:Fc in healthy humans [35]. To fully demonstrate the benefits of f-652, we used two different doses of LPS to cause ALI of varying injury severity.

Treatment of LPS injured mice with fk-652 led to decreased inflammation in the lungs as demonstrated by less cellular influx. Similarly, when looking at inflammatory cytokines in the BAL after lung injury, we found that IL-22:Fc resulted in a blunted inflammatory response. Interleukin-6 and TNF- α are well-known mediators of inflammation in ARDS [36, 37]. Both of these inflammatory mediators were found to be decreased in IL-22:Fc treated mice after LPS injury. Our findings are consistent with previous studies that have showed decreased total cell counts, neutrophils, lymphocytes, and macrophages in the BAL of mice on a pro-IL-22 genetic setting after influenza injury. This same study showed that increased IL-22 lead to decreased expression of pro-inflammatory mediators such as IL-6 and IFN- γ [38]. In a sterile, bleomycin-induced lung injury model, Sonnenberg et al. demonstrated that IL-22 decreases the cellular influx into the lungs. Our study is the first to demonstrate that fk-652 may have therapeutic potential in an animal model of ARDS. It is important to note that the present study looked at the role of IL-22 in isolated LPS lung injury. Another study has shown that IL-22 is pathogenic in a murine model of systemic endotoxemia. This study showed that IL-22 deficient mice are resistant to LPS-induced mortality [39]. This was not observed in our model of LPS lung injury and may be a reflection of the two different animal models. Further studies are need to elucidate the negative effects of IL-22 in systemic LPS toxemia.

Our group’s previous work showed that IL-22 results in less protein leak into the lung after influenza injury due to promotion of tight junction formation in the epithelial layers of the lung [38]. IL-22 not only helps maintain epithelial integrity in the lungs [40], but in other organ systems as well, including the kidneys and intestines [41–43]. We found that protein leak in the lung was decreased after LPS injury, although this could only be demonstrated in our low-dose LPS injury group. While this decreased protein leak in the IL-22:Fc treated animals is likely in part to improved integrity of the epithelial layer of the alveoli, we also demonstrated that IL-22:Fc helps maintain the endothelial glycocalyx after low-dose LPS injury (Fig 5). The endothelial glycocalyx (EG) is a glycoprotein matrix on the luminal side of endothelial cells with anti-coagulant and anti-inflammatory properties [44]. The glycocalyx plays a key role in maintaining the transvascular exchange of fluids and solutes [45]. Degradation of the glycocalyx has been implicated in the fluid and protein leak that occurs in ARDS and protection of the glycocalyx after lung injury mitigates the changes seen in the lung during ARDS [46–48]. Preservation of the glycocalyx can occur by suppression of metalloproteinases or heparinases or by induction of the biosynthesis of the glycoprotein layer. Further studies are needed to determine the mechanism by which IL-22 has its pro-glycocalyx effect. Previous work has shown that IL-22:Fc may protect the endothelial glycocalyx through reduced expression of matrix metalloproteinases [49]. While we did not demonstrate decreased protein leak in the high-dose LPS injured animals, this may be due to the time course in which protein leak was examined. Examining degree of protein leak closer to injury or after a longer period of recovery may demonstrate improved trans-capillary leak in IL-22:Fc treated animals. Decreased protein BAL was observed in the low-dose injured animals suggesting that IL-22:Fc ameliorates protein leakage in acute lung injury.

Endogenous IL-22 plays a role in the intrinsic repair of the injured lung and in clearance of infection [20, 24]. We attempted to determine if endogenous IL-22 plays a role in the anti-inflammatory effects seen with fk-652 administration after LPS injury. Because fk-652 is a human protein, we are able to differentiate the human IL-22:Fc protein from endogenous, murine IL-22. Human IL-22:Fc was found in the BAL fluid of treated animals (Fig 6A and 6B), suggesting that the fk-652 protein reaches the injured lungs and is responsible for a beneficial effect. In the low-dose LPS group, endogenous mouse IL-22 in the BAL was found in higher concentrations than sham animals (Fig 6D). This suggests that fk-652 encourages endogenous secretion of IL-22. High-dose LPS injured animals had higher levels of mouse IL-22 found in BAL, suggesting that secretion of IL-22 occurs in proportion to lung injury.

The IL-22Ra1 receptor plays a critical role in IL-22 ability to help clear viral and bacterial lung infection [24, 50]. We demonstrated that IL-22Ra1 receptors are present on alveoli after LPS lung injury. Treatment with IL-22:Fc did not result in a change in expression of the IL-22Ra1 receptor. Our data also indicates that activation of IL-22:FcRa1 by IL-22:Fc alleviated acute lung injury through activation of STAT-3. The integral role of the phosphorylated-STAT-3 pathway in repair from acute lung injury has been described in prior studies [51, 52]. The IL-22Ra1 receptor is known to help clear lung bacterial infection via the phosphorylated-STAT-3 pathway [53]. Prior research has shown that IL-22Ra1 receptors on the liver play a key role in clearance of bacterial pneumonia from the lung. Mice with a hepatic specific deletion of the IL-22Ra1 receptor had higher burdens of pneumococcal infection. In addition, mice with pneumococcal infection treated with IL-22:Fc had decreased infection burden in both the lung and liver, with increased C3 binding. This suggests IL-22:Fc may decrease bacterial burden through increased hepatic expression of C3. While we did not examine hepatic signaling in our study, future research is needed to determine if this hepatic signaling of IL-22:Fc leads to improved results in a non-infectious model of LPS acute lung injury.

RNAseq demonstrated decreased transcription of pro-inflammatory proteins Interleukin-12a (IL-12a) and Macrophage Inflammatory Protein-1β (CCL4). Decreased CCL4 in the BAL of high-dose LPS injured mice was confirmed in those treated with IL-22:Fc. While interleukin-12a levels were not measured in this study, it is a pro-inflammatory and immunomodulatory cytokine and decreased expression by RNAseq in IL-22:Fc treated animals is consistent with our overall findings [54]. CCL4 is a major factor produced by macrophages and monocytes after LPS exposure [55]. CCL4 has a strong inflammatory and chemotactic effect and anti-inflammatory effects seen with IL-22:Fc treatment may in part be due to its decreased expression. RNAseq also demonstrated increased expression of several extracellular matrix-receptor interactions, including Tenascin C (Tnc), collagen, type I, alpha 1 (COL1a1), collagen, type VI, alpha 3 (Col6a3), and collagen, type I, alpha 2 (Col1a2) expression. Collagen, type I, alpha 1 and type I, alpha 2 are important extracellular matrix components in the repair process of the lung after LPS injury [56, 57]. The prevalence of these genes in the presence of a decrease in inflammatory mediators seen in the IL-22:Fc treated animals suggests that the injured lungs have moved on from an inflammatory stage to a reparative stage. Further studies are needed to better characterize how IL-22:Fc affects extracellular matrix deposition after acute lung injury.

This study was not without limitations. IL-22:Fc were given to animals 30 minutes after injury. This was to establish proof of concept that IL-22:Fc may have a therapeutic benefit in a pre-clinical model of ARDS. Further studies are needed to determine how long after injury and at what dose IL-22:Fc may have a beneficial effect. In addition, we carried out sacrifice of animals 4 days after injury. Longer-term survival studies are necessary to determine the full effects of IL-22:Fc after lung injury. IL-22:Fc can have pro- or anti-inflammatory effects based on organ tissue or disease process [58]. Further studies are needed to determine which patient populations may benefit from IL-22:Fc.

## Conclusions

IL-22:Fc leads to decreased inflammation and protein leak in a pre-clinical model of LPS acute lung injury. In addition, IL-22:Fc preserves the endothelial glycocalyx and leads to increased endogenous IL-22 production. These findings suggest a potential therapeutic effect of Fk-652 in ARDS.

## Supporting information

S1 File (ZIP)

## References

[1] Watkins TR, Nathens AB, Cooke CR, Psaty BM, Maier RV, Cuschieri J, et al. Acute respiratory distress syndrome after trauma: development and validation of a predictive model. Crit Care Med. 2012;40(8):2295–303. doi: 10.1097/CCM.0b013e3182544f6a 22809905 PMC3400931

[2] Kamdar BB, Huang M, Dinglas VD, Colantuoni E, von Wachter TM, Hopkins RO, et al. Joblessness and Lost Earnings after Acute Respiratory Distress Syndrome in a 1-Year National Multicenter Study. Am J Respir Crit Care Med. 2017;196(8):1012–20. doi: 10.1164/rccm.201611-2327OC 28448162 PMC5649982

[3] Pais FM, Sinha P, Liu KD, Matthay MA. Influence of Clinical Factors and Exclusion Criteria on Mortality in ARDS Observational Studies and Randomized Controlled Trials. Respir Care. 2018;63(8):1060–9. doi: 10.4187/respcare.06034 29991643

[4] Khandelwal N, Hough CL, Bansal A, Veenstra DL, Treggiari MM. Long-term survival in patients with severe acute respiratory distress syndrome and rescue therapies for refractory hypoxemia*. Crit Care Med. 2014;42(7):1610–8. doi: 10.1097/CCM.0000000000000322 24732240 PMC4061153

[5] Adhikari NKJ, McAndrews MP, Tansey CM, Matte A, Pinto R, Cheung AM, et al. Self-reported symptoms of depression and memory dysfunction in survivors of ARDS. Chest. 2009;135(3):678–87. doi: 10.1378/chest.08-0974 19265087 PMC5233444

[6] Nelson BJ, Weinert CR, Bury CL, Marinelli WA, Gross CR. Intensive care unit drug use and subsequent quality of life in acute lung injury patients. Critical care medicine. 2000;28(11):3626–30. doi: 10.1097/00003246-200011000-00013 11098964

[7] Herridge MS, Tansey CM, Matte A, Tomlinson G, Diaz-Granados N, Cooper A, et al. Functional disability 5 years after acute respiratory distress syndrome. N Engl J Med. 2011;364(14):1293–304. doi: 10.1056/NEJMoa1011802 21470008

[8] Guerin C, Reignier J, Richard JC, Beuret P, Gacouin A, Boulain T, et al. Prone positioning in severe acute respiratory distress syndrome. N Engl J Med. 2013;368(23):2159–68. doi: 10.1056/NEJMoa1214103 23688302

[9] Network ARDS. Ventilation with lower tidal volumes as compared with traditional tidal volumes for acute lung injury and the acute respiratory distress syndrome. New Engl J Med. 2000;342(18):1301–8. doi: 10.1056/NEJM200005043421801 10793162

[10] Papazian L, Forel JM, Gacouin A, Penot-Ragon C, Perrin G, Loundou A, et al. Neuromuscular blockers in early acute respiratory distress syndrome. N Engl J Med. 2010;363(12):1107–16. doi: 10.1056/NEJMoa1005372 20843245

[11] Combes A, Hajage D, Capellier G, Demoule A, Lavoue S, Guervilly C, et al. Extracorporeal Membrane Oxygenation for Severe Acute Respiratory Distress Syndrome. N Engl J Med. 2018;378(21):1965–75. doi: 10.1056/NEJMoa1800385 29791822

[12] Gessner MA, Werner JL, Lilly LM, Nelson MP, Metz AE, Dunaway CW, et al. Dectin-1-dependent interleukin-22 contributes to early innate lung defense against Aspergillus fumigatus. Infect Immun. 2012;80(1):410–7. doi: 10.1128/IAI.05939-11 22038916 PMC3255669

[13] Public Law 104–208, (1997).

[14] Peng Y, Gao XL, Yang J, Shekhar S, Wang SH, Fan YJ, et al. Interleukin-22 Promotes T Helper 1 (Th1)/Th17 Immunity in Chlamydial Lung Infection. Mol Med. 2014;20(1):109–19. 24531835 10.2119/molmed.2013.00115 PMC3960397

[15] Van Maele L, Carnoy C, Cayet D, Ivanov S, Porte R, Deruy E, et al. Activation of Type 3 innate lymphoid cells and interleukin 22 secretion in the lungs during Streptococcus pneumoniae infection. J Infect Dis. 2014;210(3):493–503. doi: 10.1093/infdis/jiu106 24577508

[16] Xu X, Weiss ID, Zhang HWH, Singh SP, Wynn TA, Wilson MS, et al. Conventional NK Cells Can Produce IL-22 and Promote Host Defense in Klebsiella pneumoniae Pneumonia. Journal of Immunology. 2014;192(4):1778–86. doi: 10.4049/jimmunol.1300039 24442439 PMC3995347

[17] Pociask D, Yan X, Kolls J. IL-22 reduces the pulmonary injury and lethality of influenza infection (CCR4P. 201). Am Assoc Immnol; 2015.

[18] Starkey MR, Plank MW, Casolari P, Papi A, Pavlidis S, Guo Y, et al. IL-22 and its receptors are increased in human and experimental COPD and contribute to pathogenesis. Eur Respir J. 2019;54(1). doi: 10.1183/13993003.00174-2018 31196943 PMC8132110

[19] Broquet A, Besbes A, Martin J, Jacqueline C, Vourc’h M, Roquilly A, et al. Interleukin-22 regulates interferon lambda expression in a mice model of pseudomonas aeruginosa pneumonia. Mol Immunol. 2020;118:52–9. doi: 10.1016/j.molimm.2019.12.003 31855807

[20] Pociask DA, Scheller EV, Mandalapu S, McHugh KJ, Enelow RI, Fattman CL, et al. IL-22 is essential for lung epithelial repair following influenza infection. The American journal of pathology. 2013;182(4):1286–96. doi: 10.1016/j.ajpath.2012.12.007 23490254 PMC3620404

[21] Wang X, Ota N, Manzanillo P, Kates L, Zavala-Solorio J, Eidenschenk C, et al. Interleukin-22 alleviates metabolic disorders and restores mucosal immunity in diabetes. Nature. 2014;514(7521):237–41. doi: 10.1038/nature13564 25119041

[22] Shrestha N, Lateef Z, Martey O, Bland AR, Nimick M, Rosengren R, et al. Does the mouse tail vein injection method provide a good model of lung cancer? F1000Research. 2019;8. doi: 10.12688/f1000research.17047.1 31448098 PMC6668049

[23] Goula D, Benoist C, Mantero S, Merlo G, Levi G, Demeneix BA. Polyethylenimine-based intravenous delivery of transgenes to mouse lung. Gene Ther. 1998;5(9):1291–5. doi: 10.1038/sj.gt.3300717 9930332

[24] Trevejo-Nunez G, Elsegeiny W, Conboy P, Chen K, Kolls JK. Critical Role of IL-22/IL22-RA1 Signaling in Pneumococcal Pneumonia. J Immunol. 2016;197(5):1877–83. doi: 10.4049/jimmunol.1600528 27456484 PMC4992592

[25] Draghici S, Khatri P, Tarca AL, Amin K, Done A, Voichita C, et al. A systems biology approach for pathway level analysis. Genome Res. 2007;17(10):1537–45. doi: 10.1101/gr.6202607 17785539 PMC1987343

[26] Donato M, Xu Z, Tomoiaga A, Granneman JG, Mackenzie RG, Bao R, et al. Analysis and correction of crosstalk effects in pathway analysis. Genome Res. 2013;23(11):1885–93. doi: 10.1101/gr.153551.112 23934932 PMC3814888

[27] Tarca AL, Draghici S, Khatri P, Hassan SS, Mittal P, Kim JS, et al. A novel signaling pathway impact analysis. Bioinformatics. 2009;25(1):75–82. doi: 10.1093/bioinformatics/btn577 18990722 PMC2732297

[28] Ahsan S, Drăghici S. Identifying significantly impacted pathways and putative mechanisms with iPathwayGuide. Current protocols in bioinformatics. 2017;57(1):7.15. 1–7. 30. doi: 10.1002/cpbi.24 28654712

[29] Love MI, Huber W, Anders S. Moderated estimation of fold change and dispersion for RNA-seq data with DESeq2. Genome biology. 2014;15(12):1–21. doi: 10.1186/s13059-014-0550-8 25516281 PMC4302049

[30] Kramer A, Green J, Pollard J Jr., Tugendreich S. Causal analysis approaches in Ingenuity Pathway Analysis. Bioinformatics. 2014;30(4):523–30. doi: 10.1093/bioinformatics/btt703 24336805 PMC3928520

[31] Warnes GR, Bolker B, Bonebakker L, Gentleman R, Huber W, Liaw A, et al. gplots: Various R programming tools for plotting data. R package version. 2009;2(4):1.

[32] Livak KJ, Schmittgen TD. Analysis of relative gene expression data using real-time quantitative PCR and the 2− ΔΔCT method. methods. 2001;25(4):402–8. doi: 10.1006/meth.2001.1262 11846609

[33] Howard BM, Kornblith LZ, Hendrickson CM, Redick BJ, Conroy AS, Nelson MF, et al. Differences in degree, differences in kind: characterizing lung injury in trauma. J Trauma Acute Care Surg. 2015;78(4):735–41. doi: 10.1097/TA.0000000000000583 25742257 PMC4624341

[34] Robles AJ, Kornblith LZ, Hendrickson CM, Howard BM, Conroy AS, Moazed F, et al. Health care utilization and the cost of post-traumatic ARDS care. The journal of trauma and acute care surgery. 2018;85(1):148. doi: 10.1097/TA.0000000000001926 29958249 PMC6029709

[35] Tang KY, Lickliter J, Huang ZH, Xian ZS, Chen HY, Huang C, et al. Safety, pharmacokinetics, and biomarkers of F-652, a recombinant human interleukin-22 dimer, in healthy subjects. Cell Mol Immunol. 2019;16(5):473–82. doi: 10.1038/s41423-018-0029-8 29670279 PMC6474205

[36] Neville LF, Abdullah F, McDonnell PM, Young PR, Feuerstein GZ, Rabinovici R. Mob-1 expression in IL-2-induced ARDS: regulation by TNF-alpha. Am J Physiol. 1995;269(6 Pt 1):L884–90. doi: 10.1152/ajplung.1995.269.6.L884 8572251

[37] Oliveira MC Jr, Greiffo FR, Rigonato-Oliveira NC, Custódio RWA, Silva VR, Damaceno-Rodrigues NR, et al. Low level laser therapy reduces acute lung inflammation in a model of pulmonary and extrapulmonary LPS-induced ARDS. Journal of Photochemistry and Photobiology B: Biology. 2014;134:57–63. 10.1016/j.jphotobiol.2014.03.021 24792475

[38] Hebert KD, McLaughlin N, Galeas-Pena M, Zhang Z, Eddens T, Govero A, et al. Targeting the IL-22/IL-22BP axis enhances tight junctions and reduces inflammation during influenza infection. Mucosal Immunol. 2020;13(1):64–74. doi: 10.1038/s41385-019-0206-9 31597930 PMC6917921

[39] Dumoutier L, De Heusch M, Orabona C, Satoh‐Takayama N, Eberl G, Sirard JC, et al. IL‐22 is produced by γC‐independent CD25+ CCR6+ innate murine spleen cells upon inflammatory stimuli and contributes to LPS‐induced lethality. European journal of immunology. 2011;41(4):1075–85. doi: 10.1002/eji.201040878 21400499

[40] Alcorn JF. IL-22 Plays a Critical Role in Maintaining Epithelial Integrity During Pulmonary Infection. Front Immunol. 2020;11:1160. doi: 10.3389/fimmu.2020.01160 32582219 PMC7296169

[41] Lo BC, Shin SB, Canals Hernaez D, Refaeli I, Yu HB, Goebeler V, et al. IL-22 Preserves Gut Epithelial Integrity and Promotes Disease Remission during Chronic Salmonella Infection. J Immunol. 2019;202(3):956–65. doi: 10.4049/jimmunol.1801308 30617224

[42] Weidenbusch M, Song S, Iwakura T, Shi C, Rodler S, Kobold S, et al. IL-22 sustains epithelial integrity in progressive kidney remodeling and fibrosis. Physiol Rep. 2018;6(16):e13817. doi: 10.14814/phy2.13817 30156011 PMC6113136

[43] Zindl CL, Lai JF, Lee YK, Maynard CL, Harbour SN, Ouyang W, et al. IL-22-producing neutrophils contribute to antimicrobial defense and restitution of colonic epithelial integrity during colitis. Proc Natl Acad Sci U S A. 2013;110(31):12768–73. doi: 10.1073/pnas.1300318110 23781104 PMC3732935

[44] Chelazzi C, Villa G, Mancinelli P, De Gaudio AR, Adembri C. Glycocalyx and sepsis-induced alterations in vascular permeability. Crit Care. 2015;19(1):26. doi: 10.1186/s13054-015-0741-z 25887223 PMC4308932

[45] Rehm M, Zahler S, Lötsch M, Welsch U, Conzen P, Jacob M, et al. Endothelial glycocalyx as an additional barrier determining extravasation of 6% hydroxyethyl starch or 5% albumin solutions in the coronary vascular bed. Anesthesiology: The Journal of the American Society of Anesthesiologists. 2004;100(5):1211–23. doi: 10.1097/00000542-200405000-00025 15114220

[46] Murphy LS, Wickersham N, McNeil JB, Shaver CM, May AK, Bastarache JA, et al. Endothelial glycocalyx degradation is more severe in patients with non-pulmonary sepsis compared to pulmonary sepsis and associates with risk of ARDS and other organ dysfunction. Annals of Intensive Care. 2017;7(1):1–9. doi: 10.1186/s13613-016-0221-x 28986821 PMC5630541

[47] Kong G, Huang X, Wang L, Li Y, Sun T, Han S, et al. Astilbin alleviates LPS-induced ARDS by suppressing MAPK signaling pathway and protecting pulmonary endothelial glycocalyx. Int Immunopharmacol. 2016;36:51–8. doi: 10.1016/j.intimp.2016.03.039 27111514

[48] Wang L, Huang X, Kong G, Xu H, Li J, Hao D, et al. Ulinastatin attenuates pulmonary endothelial glycocalyx damage and inhibits endothelial heparanase activity in LPS-induced ARDS. Biochem Biophys Res Commun. 2016;478(2):669–75. doi: 10.1016/j.bbrc.2016.08.005 27498004

[49] Taghavi S, Abdullah S, Duchesne J, Pociask D, Kolls J, Jackson-Weaver O. Interleukin 22 mitigates endothelial glycocalyx shedding after lipopolysaccharide injury. J Trauma Acute Care Surg. 2021;90(2):337–45. doi: 10.1097/TA.0000000000003019 33502147 PMC7872437

[50] Hebert KD, Mclaughlin N, Zhang Z, Cipriani A, Alcorn JF, Pociask DA. IL-22Ra1 is induced during influenza infection by direct and indirect TLR3 induction of STAT1. Resp Res. 2019;20(1):1–10. doi: 10.1186/s12931-019-1153-4 31416461 PMC6694528

[51] Gao H, Guo RF, Speyer CL, Reuben J, Neff TA, Hoesel LM, et al. Stat3 activation in acute lung injury. J Immunol. 2004;172(12):7703–12. doi: 10.4049/jimmunol.172.12.7703 15187153

[52] Zhao J, Yu H, Liu Y, Gibson SA, Yan Z, Xu X, et al. Protective effect of suppressing STAT3 activity in LPS-induced acute lung injury. Am J Physiol Lung Cell Mol Physiol. 2016;311(5):L868–L80. doi: 10.1152/ajplung.00281.2016 27638904 PMC5130536

[53] Aujla SJ, Chan YR, Zheng M, Fei M, Askew DJ, Pociask DA, et al. IL-22 mediates mucosal host defense against Gram-negative bacterial pneumonia. Nat Med. 2008;14(3):275–81. doi: 10.1038/nm1710 18264110 PMC2901867

[54] Trinchieri G. Interleukin-12: A cytokine at the interface of inflammation and immunity. Advances in Immunology, Vol 70. 1998;70:83–243. doi: 10.1016/s0065-2776(08)60387-9 9755338

[55] Sherry B, Tekamp-Olson P, Gallegos C, Bauer D, Davatelis G, Wolpe S, et al. Resolution of the two components of macrophage inflammatory protein 1, and cloning and characterization of one of those components, macrophage inflammatory protein 1 beta. The Journal of experimental medicine. 1988;168(6):2251–9. doi: 10.1084/jem.168.6.2251 3058856 PMC2189160

[56] de Souza Xavier Costa N, Ribeiro Junior G, Dos Santos Alemany AA, Belotti L, Zati DH, Frota Cavalcante M, et al. Early and late pulmonary effects of nebulized LPS in mice: An acute lung injury model. PLoS One. 2017;12(9):e0185474. doi: 10.1371/journal.pone.0185474 28953963 PMC5617199

[57] Marshall R, Bellingan G, Laurent G. The acute respiratory distress syndrome: fibrosis in the fast lane. BMJ Publishing Group Ltd; 1998. 10.1136/thx.53.10.815 PMC1745098 10193364

[58] Shabgah AG, Navashenaq JG, Shabgah OG, Mohammadi H, Sahebkar A. Interleukin-22 in human inflammatory diseases and viral infections. Autoimmun Rev. 2017;16(12):1209–18. doi: 10.1016/j.autrev.2017.10.004 29037907

